# The combined toxicological effects of titanium dioxide nanoparticles and bisphenol A on zebrafish embryos

**DOI:** 10.1186/1556-276X-9-406

**Published:** 2014-08-20

**Authors:** Jun Yan, Bencheng Lin, Chuanlu Hu, Huashan Zhang, Zhiqing Lin, Zhuge Xi

**Affiliations:** 1 Tianjin Institute of Health and Environmental Medicine, #55 Munan Road, Heping District, Tianjin 300050, China

**Keywords:** TiO_2_-NPs, BPA, Combined toxicological effects, Zebrafish embryo, Adsorption

## Abstract

Environmental pollutants co-exist and exhibit interaction effects that are different from those associated with a single pollutant. As one of the more commonly manufactured nanomaterials, titanium dioxide nanoparticles (TiO_2_-NPs) are most likely to bind to other contaminants in water. In this paper, we aimed to study the combined toxicological effects of TiO_2_-NPs and bisphenol A (BPA) on organism. First, *in vitro* adsorption experiments were conducted to determine the adsorptive interaction between TiO_2_-NPs and BPA. Second, zebrafish embryo toxicity tests were performed to monitor for changes in the toxicological effects associated with the two chemicals. The study results demonstrated that adsorptive interactions exist between the two chemicals and increased toxicity effects which included an advanced toxicological effect time, decreased survival, increased morphological abnormalities, and delayed embryo hatching. Also, we suggest that the mode of combined action has a synergistic effect. Based on this, we postulate that concomitant exposure to TiO_2_-NPs and BPA increased BPA bioavailability and uptake into cells and organisms. Further studies are required to understand the mechanisms of interactions of this mixture.

## Background

Environmental pollutants co-exist and exhibit interaction effects. This interaction effect is influenced by not only the form and distribution of the pollutants between media and affected organisms but also transport and biotransformation [1,2], which may therefore change the toxicological effects on organisms. Therefore, it is necessary to examine the toxicological effects associated with two or more co-existing compounds.

As we have known, titanium dioxide nanoparticles (TiO_2_-NPs) have been extensively used in industrial production as well as scientific, biological, and medical fields. TiO_2_-NPs can be released into the environment by a variety of pathways, and the ultimate destination would be surface water. In recent years, TiO_2_-NPs have been identified in surface runoff and wastewater [3-5]. There is emerging literature on the ecotoxicity of nanosized TiO_2_[6-8]. But one of the significant issues is that TiO_2_-NPs are most likely to adsorb other organic contaminants in water based on their exceptional physicochemical properties, such as small size, high surface-to-mass ratio, and greater reactivity. The possible interaction of TiO_2_-NPs with other toxicants has been one of the hot topics in nanotoxicology.

Some researchers have reported on the adsorption of carbon nanotubes [9-18]. Intermittent articles have studied about the adsorption of metal elements onto TiO_2_-NPs [19,20]. Although previous studies have proven an adsorption interaction between nanomaterials (NMs) and organic pollutants, too less data are available on their combined biological toxic effects *in vivo* and the possible toxicological change of organic pollutants adsorbed by NMs.

Bisphenol A (4,4′-isopropylidenediphenol, BPA) is widely used as a key raw material in the manufacture of polycarbonate plastic and epoxy resins. BPA can be present even in treated effluent after wastewater treatment processes [21]. BPA has limited biodegradation under anaerobic conditions [22]. Aquatic organisms near BPA output point sources are at the greatest risk of the harmful effects of BPA [23,24].

As an alternative to acute fish toxicity testing, the zebrafish embryo test has proven to be more sensitive than the fish cytotoxicity assay [25]. Upon comparing the early embryonic stages of other Organisation for Economic Co-operation and Development (OECD)-recommended species, such as the fathead minnow and the Japanese medaka, zebrafish appeared to be the best model for routine embryo toxicity testing, and the zebrafish embryo assay is a promising tool to replace the acute fish toxicity test [26,27].

In the present study, we chose BPA as a representative organic compound and studied the toxicological effects associated with TiO_2_-NPs by using a zebrafish embryo model. The study consisted of the following two parts: first, *in vitro* adsorption experiments were performed to determine the adsorptive interaction between TiO_2_-NPs and BPA; second, zebrafish embryo toxicity tests were performed to monitor changes in the toxicological effects of the two chemicals. We expect that the study results will be useful for more accurate risk assessment of NMs and organic pollutants in environments. We focus on the issue of potential environmental risks; we aim to study the combined toxicological effects of TiO_2_-NPs and BPA on organism.

## Methods

### Chemicals

**Figure 1:**
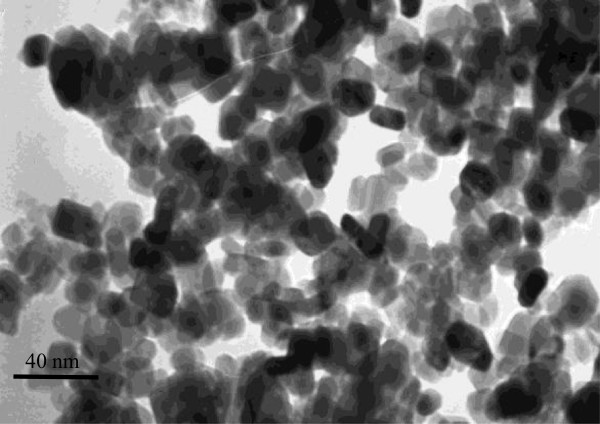
**Particle characterization of the TiO**_**2**_**-NPs suspension sample.**

TiO_2_-NPs (<25 nm; purity ≥99.7%; anatase) were purchased from Sigma-Aldrich Co. (St. Louis, MO, USA). The particles were prepared in dilution water (294.0 mg/L CaCl_2_ · 2H_2_O; 123.3 mg/L MgSO_4_ · 7H_2_O; 63.0 mg/L NaHCO_3_; 5.5 mg/L KCl [28]) by vortexing the suspension ten times for 10 s followed by sonication for 30 min in a bath-type sonicator (35-kHz frequency, Fisherbrand FB 11010, Shanghai, China) to break down agglomerates and ensure a uniform suspension. Particle characterization of the TiO_2_-NPs suspension sample was examined by a transmission electron microscope (TEM; JEM-2010FEF, JEOL, Akishima-shi, Japan) (Figure 1).

Bisphenol A (BPA; C_15_H_16_O_2_, purity >99.5%) was purchased from the Chemical Agent Co. (Tianjin, China). A BPA stock solution was prepared in an aqueous solution that contained ethanol as a co-solvent. The final concentration of ethanol was less than 1%, and our previous evaluations had demonstrated no adverse biological effect at this concentration. The stock solution was then diluted to specific test concentrations with the dilution solvent.

### Adsorption experiments of BPA on TiO_2_-NPs *in vitro*: analysis of BPA concentration before and after mixture

To analyze if there were changes of BPA concentration after mixing with TiO_2_-NPs, different concentrations of BPA solutions (5 and 10 mg/L) and combined solutions (BPA 5 mg/L + TiO_2_ 10 mg/L, BPA 5 mg/L + TiO_2_ 50 mg/L, BPA 10 mg/L + TiO_2_ 10 mg/L, and BPA 10 mg/L + TiO_2_ 50 mg/L) were prepared. Then the combined solutions were added to 50-mL plastic centrifuge tubes (in a preliminary experiment, the plastic centrifuge tubes were proven to have no effect on the BPA concentration). Subsequently, the tubes were centrifuged for 10 min at 14,000 rpm using a high-speed centrifuge (HIMAC CR 22G II, Hitachi, Ltd., Chiyoda-ku, Japan), and the supernatant was collected and centrifuged again at 14,000 rpm. Before injection, the BPA solutions and the combined solutions were passed through 0.22-μm filter membranes, respectively. The samples were analyzed by high-performance liquid chromatography (Waters 2695 HPLC, Waters Corp., Milford, MA, USA) with a photodiode array detector (Waters 2998 PDA) for BPA levels. The samples were separated on a CAPCELL PAK C_18_ column (4.6 × 150 mm, 5 μm, Waters Corp.) using a mobile phase of 60% acetonitrile and 40% water at a flow rate of 1.0 mL/min. The injection volume was 10 μL, and the column temperature was 20°C. The pH values for all of the samples before and after the adsorption experiments were similar and were approximately neutral.

### Adsorption experiments of BPA on TiO_2_-NPs *in vitro*: adsorption kinetics of BPA on TiO_2_-NPs

A solution of BPA 5 mg/L + TiO_2_ 10 mg/L was used to measure adsorption kinetics. The combined treatment solution was shaken at 250 rpm in a reciprocating shaker at 20°C ± 1°C. The kinetic data were collected with an initial BPA concentration of 5 mg/L. Subsequently, 10 mL of the treatment solution was collected and centrifuged twice at 14,000 rpm at 0, 5, 10, 30, 60, 90, 120, and 180 min, and the BPA concentrations were analyzed.

### Zebrafish maintenance and embryo collection

Juvenile zebrafish were purchased from a local aquarium in Tianjin, China. The fish were kept at aquarium conditions of 26°C ± 1°C at a density of ≤1/L using charcoal-filtered tap water. The pH of the maintenance water was within the range of 6.8 to 8.4. Oxygen saturation in the maintenance aquaria was kept above 80%. The fish were maintained with a 14:10-h light/dark cycle and fed dry flake food and frozen midge larvae twice or thrice per day.

The fish were bred for at least 6 months, and females and males were continuously maintained in a ratio of 1:2. The mature zebrafish that were used for egg production were free of macroscopically discernible symptoms of infection and disease. Whenever eggs were required, several spawning traps covered with stainless steel mesh were placed on the bottom of the aquaria in the evening, and eggs were collected the following morning. Spawning and fertilization were initiated by rapidly illuminating the aquaria, which was terminated 1 h later by removing the spawning traps. The fish eggs were collected and rinsed three times in dilution water to remove any residue on the egg’s surface. Subsequently, the eggs were immediately exposed to different treatment solutions. Fertilized and normally developing embryos were selected under a stereomicroscope (×8 to × 50) at 4 h post-fertilization (hpf) (i.e., the sphere stage of the blastula period) and used for exposure experiments.

### Single TiO_2_-NPs exposure to zebrafish embryos

To determine the concentration of TiO_2_ in the associated toxicological exposure, we first studied the effect of TiO_2_-NPs exposure on zebrafish embryo development. The concentration series of TiO_2_-NPs suspensions were 0, 2.5, 5, 10, 20, and 40 mg/L. These test solutions were prepared by diluting a stock solution of 40 mg/L TiO_2_-NPs. TiO_2_-NPs suspensions were freshly prepared before the fish eggs were exposed.

### Mixture exposure of TiO_2_-NPs and BPA to zebrafish embryos

The associated toxicity test in this study consisted of five simultaneous treatment series: (a) BPA alone, (b) mixtures of BPA and TiO_2_-NPs, (c) TiO_2_-NPs alone control, (d) dilution water control, (e) dilution solvent control. Based on the effect of TiO_2_-NPs alone on zebrafish embryo development and our preliminary experiments, the exposure concentrations were determined as follows: 10 mg/L TiO_2_-NPs and different concentrations of BPA (0.5, 1, 2, 5, 10, and 20 mg/L). TiO_2_-NPs powder was weighed and added to individual BPA solutions. The mixture solutions were sonicated for 30 min and were freshly prepared before the exposure test.

### The embryo toxicity test procedure

The embryo toxicity test procedure followed the OECD guidelines for fish embryo toxicity testing [27,29]. The selected eggs were transferred to 24-well multiple-well plates with freshly prepared test solutions. In 20 wells, selected eggs were placed individually in 2 mL of the individual test solutions. The remaining 4 wells per plate were filled with 2 mL of the dilution water and one egg per well as an internal control. The pH values of the control samples were 7.8 ± 0.2. Moreover, the dilution solvent was used as a solvent control in another 24-well multiple-well plate. All of the wells were covered with a transparent plastic film and were placed on a shaker (at a speed of 40 rpm) in a climate chamber at 26°C ± 1°C with a 14:10-h light/dark cycle. Each treatment group was tested in parallel in two independent replicates, and each medium was replaced every 24 h.

The development of the embryos from blastulas to early life stages at defined times was observed with a stereomicroscope (×8 to × 50). The endpoints used for assessing developmental toxicity were recorded and described for embryos in both the control and treated groups [30]. The observation times were at 4, 8, 12, 16, 24, 36, 48, 72, and 96 hpf. Lethal and sublethal endpoints were used for determining the combined toxicological effects, including embryo survival, coagulated eggs, malformation, no extension of tail at 24 hpf, no spontaneous movements within 20 s, no heartbeat, no blood circulation and weak pigmentation, heart sac edema, spine deformation, and hatching rate.

### Determination of dispersed TiO_2_-NPs concentrations in exposure solutions

**Figure 2:**
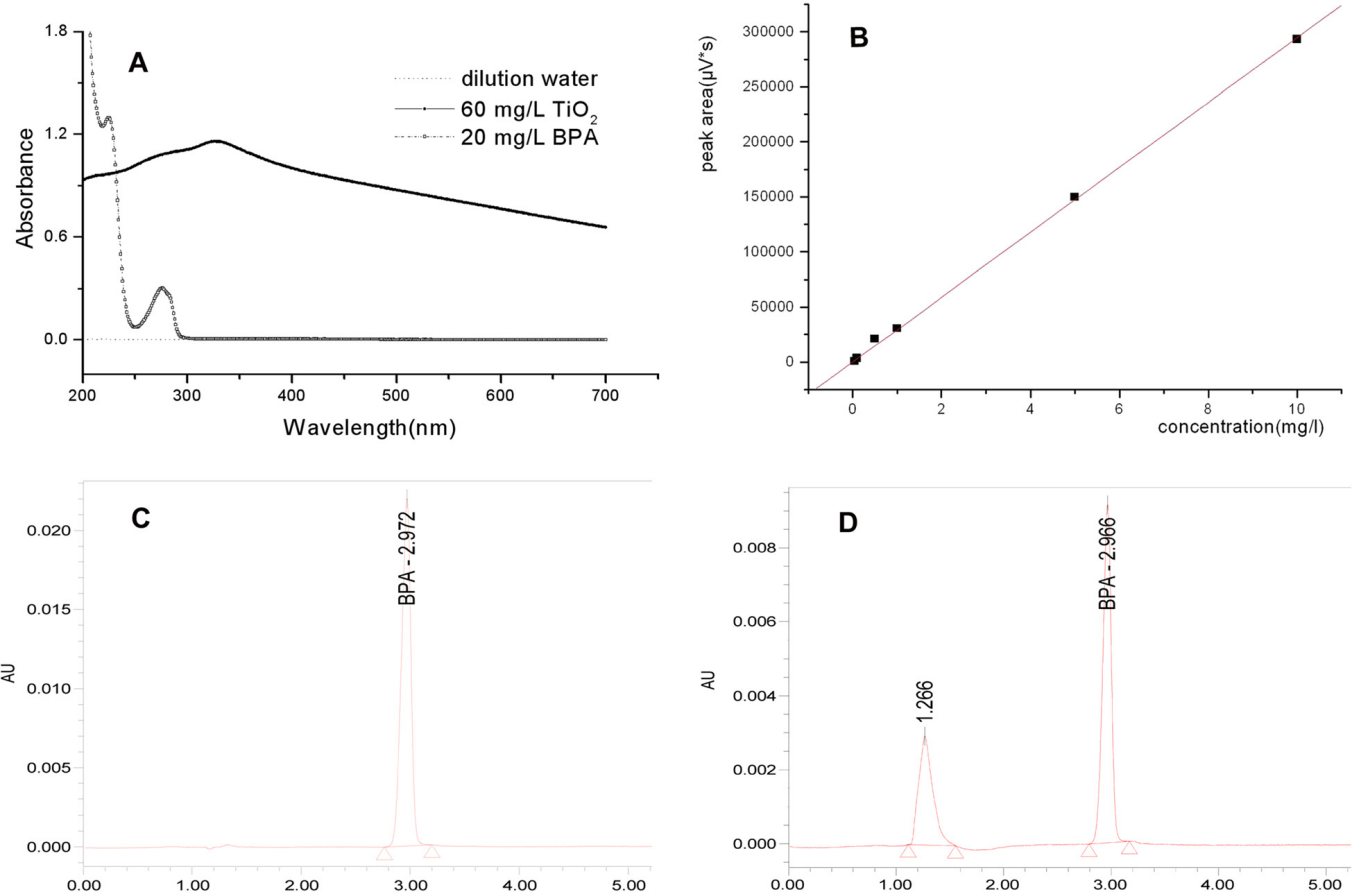
**Absorbance spectra (A), standard curve of BPA (B), and chromatograms of BPA 5 mg/L + TiO**_**2**_**10 mg/L (C, D).**

During processes of the embryo exposure, dispersed TiO_2_-NPs concentrations were monitored using an UV–VIS spectrophotometer (UV-2550, Shimadzu Corporation, Kyoto, Japan). Spectral scans of the sonicated TiO_2_-NPs suspensions (200 to 700 nm) gave the typical profile with a peak at about 329 nm. The absorbance spectra from dispersed TiO_2_-NPs are shown in Figure 2A, which shows an example of 60 mg/L TiO_2_ solution after sonicating for 30 min compared to 20 mg/L BPA solution and dilution water. Water samples were analyzed against 0 to 60 mg/L TiO_2_-NPs standards. The equation for the standard curve is *y* = 0.0149*x −* 0.0217, *r*^2^ = 0.9892. Percentages of dispersed TiO_2_-NPs concentrations at 0, 6, 12, and 24 h after dosing the embryos are shown in Table 1.

**Table 1:** **Percentages of dispersed TiO**_**2**_**-NPs concentrations**

**Exposure dose (mg/L)**	**Percentages of dispersed TiO**_**2**_**-NPs concentrations in exposure solutions (%)**			
	**0 h**	**6 h**	**12 h**	**24 h**
T2.5	99	96	93	88
T5.0	97	96	94	89
T10	99	98	92	87
T20	99	97	83	81
T40	99	97	88	79
B0.5 + T10	99	96	89	87
B1.0 + T10	99	95	90	84
B2.0 + T10	99	95	89	82
B5.0 + T10	99	98	91	85
B10 + T10	99	95	89	82
B20 + T10	99	97	91	85

### Statistical analysis

All data were obtained from the toxicological endpoints and were analyzed by type and severity. Significant differences between each exposure group and the control group were determined by one-way ANOVA within the same treatment group. For different treatments, a chi-square test was used to compare the BPA alone-exposed group with the mixture-exposed groups. A *p* value <0.05 was considered statistically significant. The graphs were compiled using ORIGIN 7.0 (OriginLab Corp., Northampton, MA, USA).

## Results

### Changes in BPA concentration before and after mixture exposure *in vitro*

In this test, we determined that the BPA concentrations of the supernatants decreased after exposure to the BPA and TiO_2_-NPs mixture. The equation for the standard curve of BPA is *Y* = 29,221.8*X* + 1945.1 (*a* = 29,221.8, *b* = 1945.1, *r*^2^ = 0.9998) (Figure 2B). Compared with the single BPA solutions, the BPA concentrations of the supernatants in the mixture solutions (BPA 5 mg/L + TiO_2_ 10 mg/L, BPA 5 mg/L + TiO_2_ 50 mg/L, BPA 10 mg/L + TiO_2_ 10 mg/L, and BPA 10 mg/L + TiO_2_ 50 mg/L) decreased by 63%, 75%, 28%, and 46%, respectively. The chromatograms of BPA 5 mg/L + TiO_2_ 10 mg/L before and after mixture exposure are shown in Figure 2C, D. This experiment primarily demonstrated that an adsorption relationship between BPA and TiO_2_-NPs did exist.

### Adsorption kinetics of BPA on TiO_2_-NPs

**Figure 3:**
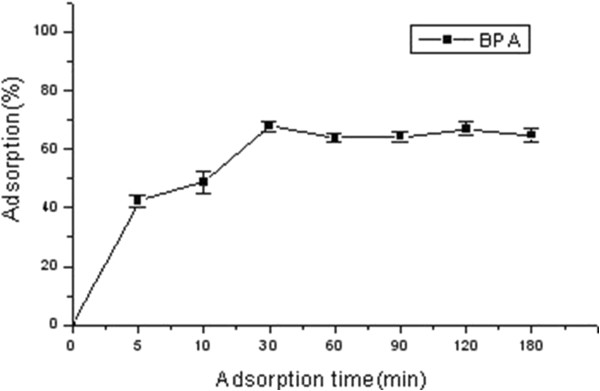
**Adsorption kinetics of BPA on TiO**_**2**_**-NPs.**

Adsorption kinetics was observed for 3 h and the results are presented in Figure 3. The initial concentration of BPA and TiO_2_-NPs was 5 and 10 mg/L, respectively. The adsorption process of BPA onto TiO_2_-NPs was fast. After the adsorption began, the adsorption percentage of BPA on TiO_2_-NPs increased rapidly and the percentage reached 40% approximately at 5 min. The maximal amount of BPA adsorbed by TiO_2_-NPs appeared at 30 min, and the value was approximately 70%. The adsorption reached equilibrium basically after 60 min.

### The effect of TiO_2_-NPs alone on zebrafish embryos

**Figure 4:**
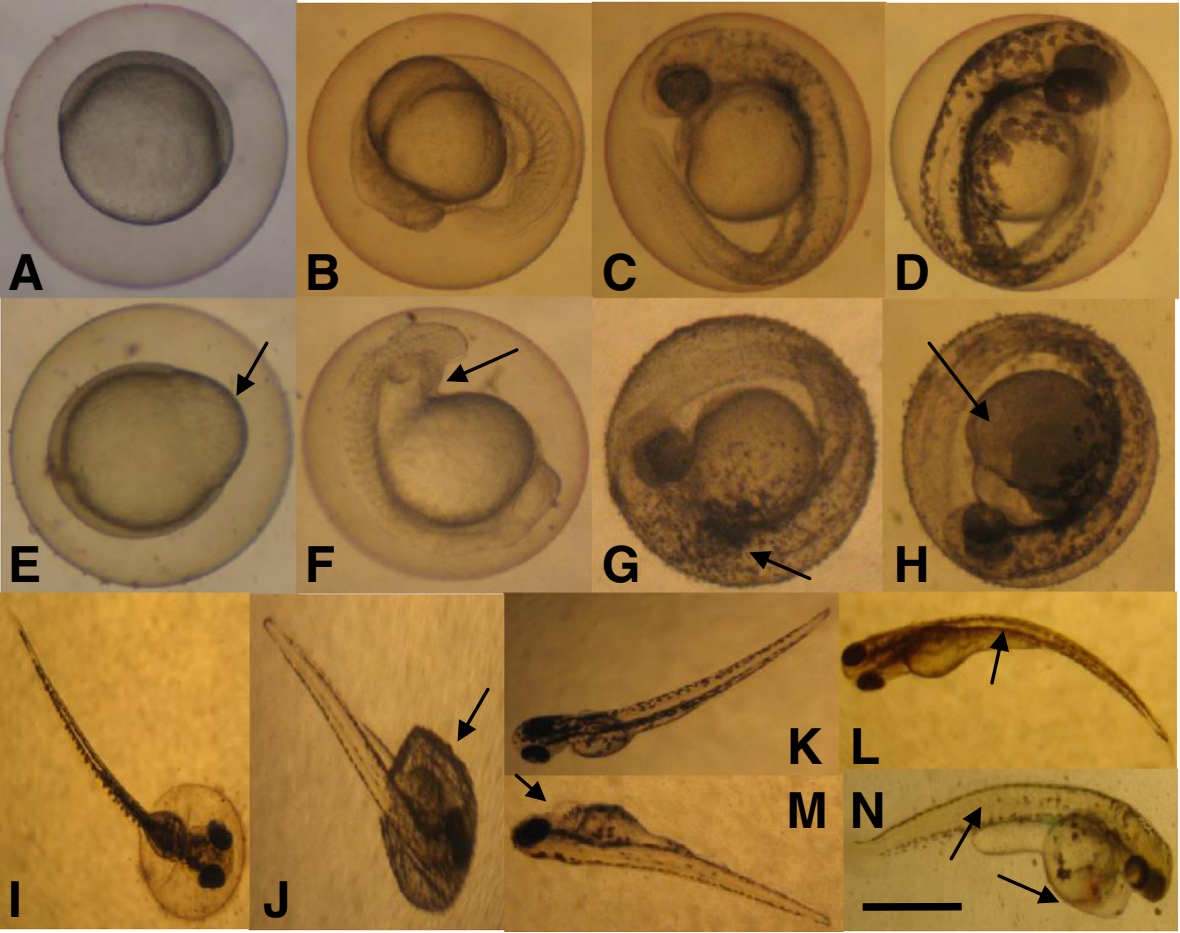
**Effect of TiO**_**2**_**-NPs alone and combined toxicological effects of TiO**_**2**_**-NPs and BPA on zebrafish embryos. (A-D, I, K)** Normal embryonic development of zebrafish. **(E, F, I-N)** Observed abnormalities (arrows). **(G, H, J)** TiO_2_-NPs accumulation (arrows) on the surface of the exposed egg envelopes. Scale bar, 385 μm in **(A)** to **(H)** and 1,050 μm in **(I)** to **(N)**.

In this study, significant morphological abnormalities were not observed in the zebrafish embryos, when exposed to TiO_2_-NPs suspensions of different concentrations. The 96-h survival rate of the embryos decreased slightly when exposed to 40 mg/L TiO_2_-NPs, but there was no significant difference between the treatment and control groups. However, TiO_2_-NPs were observed to accumulate on the surface of the exposed egg envelopes (Figure 4G, H, J). With increasing concentrations, more TiO_2_-NPs adhered to and aggregated on the surface of the egg envelopes. When the concentration was increased to 40 mg/L, the egg envelope surface became turbid and difficult to be observed.

**Figure 5:**
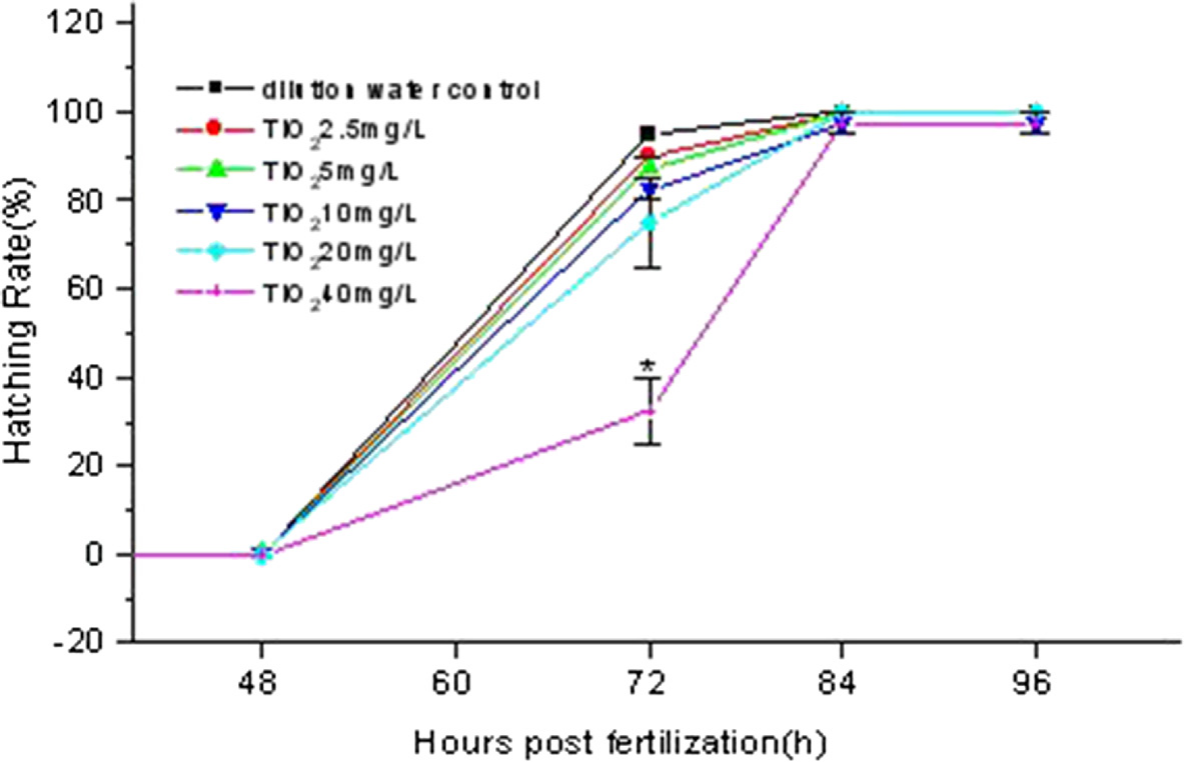
**Hatching rate of the zebrafish embryos.** *Significant difference compared to other groups (one-way ANOVA, *p* < 0.05).

Additionally, the hatching rate of the zebrafish embryos was influenced by TiO_2_-NPs exposure (Figure 5). Compared with treatment groups at lower concentrations and the control group, the hatching rate at 72 hpf of the embryos that were exposed to 40 mg/L of TiO_2_-NPs was significantly less (*p* < 0.05).

### The combined toxicological effects of TiO_2_-NPs and BPA on zebrafish embryos: embryo survival, morphological abnormalities, and hatching rate

No effect was observed in the zebrafish embryos of the dilution solvent control group (data not shown).

No dead embryos were observed in the dilution water control group. There were no significant differences between the BPA alone-exposed and mixture-exposed groups with BPA at 0.5, 1, and 2 mg/L. In addition to some abnormalities (2.5%), all of the embryos survived in the BPA alone-exposed group (5 mg/L) at 96 hpf after exposure. In contrast, all of the zebrafish embryos in the mixture-exposed groups (BPA, 5 mg/L) had died when observed at 84 hpf. Compared with the BPA alone-exposed groups, the survival rate of embryos in the mixture-exposed groups decreased. There were statistical differences between the BPA alone-exposed groups and mixture-exposed groups with BPA at 5, 10, and 20 mg/L, which occurred at 72 to 96 hpf, 48 to 72 hpf, and 48 hpf, respectively. Moreover, with the increasing doses of BPA (from 5, 10, to 20 mg/L) for the mixture-exposed groups, the survival rate of embryos showed concentration-dependent decreasing at 48 and 72 hpf (*p* < 0.05).The normal embryonic development of zebrafish at 8, 24, 36, 48, and 72 h are shown in Figure 4A, B, C, D, I, K). In this study, observed abnormalities referred to all abnormal toxicological endpoints including retarded development, for example, coagulated eggs, malformation, no extension of tail at 24 hpf, no spontaneous movements within 20 s, no heartbeat, no blood circulation and weak pigmentation, heart sac edema, spine deformation, and hatching rate. As can be seen from Figure 4, the embryos were observed as follows: developmental malformation at 8 h (e), no extension of tail at 24 h (f), spine deformation and heart sac edema and congestion at 72 h (L, M, N).

**Figure 6:**
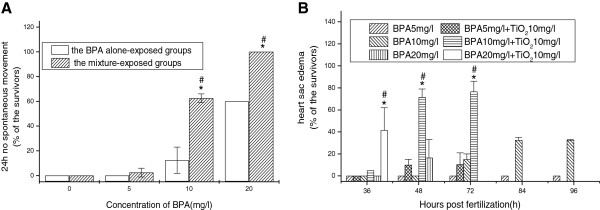
**The 24 h no spontaneous movements (A) and heart sac edema (B) of the embryos.** *Significant difference between the BPA alone-exposed and mixture-exposed groups at the same BPA dose (chi-square test, *p* < 0.05). ^#^Significant difference compared to the lower concentrations of mixture-exposed groups at the same time points (one-way ANOVA, *p* < 0.05).

There were no visible abnormal changes in addition to the hatching rate in the BPA alone-exposed groups at 0.5, 1.0, and 2.0 mg/L. Weak pigmentation at 48 hpf and spine deformation at 84 hpf were observed in the mixture-exposed groups with BPA concentrations of 0.5, 1.0, and 2.0 mg/L, but there were no significant differences between the alone- and mixture-exposed groups.With increasing concentrations of BPA, the main abnormalities were no spontaneous movements at 24 hpf and heart sac edema from 36 hpf. At 24 hpf, no spontaneous movements within 20 s of the embryos were observed in the mixture-exposed groups with BPA concentrations of 10 and 20 mg/L, which caused significant increases in the abnormality rates (i.e., 62.5% and 100%, respectively) compared with the BPA alone-exposed groups. Meanwhile, exposure to the mixture groups at 5, 10, and 20 mg/L BPA significantly increased 24 h no spontaneous movements of the embryos (Figure 6A). The embryos in the mixture-exposed groups were observed to have heart sac edema at BPA concentrations of 10 mg/L (at 48 and 72 hpf) and 20 mg/L (at 36 hpf), which caused significant increases compared with the BPA alone-exposed groups. After the mixture exposure, there were significant differences between the highest dose of mixture groups and the lower ones at the same time point, which do not conclude the death caused by mixture-exposed groups at 20 mg/L BPA from 48 hpf. It was also found that the rate of heart sac edema of the embryos exposed to the mixture groups had an increased tendency with the extension of the embryonic development time (Figure 6B).

**Figure 7:**
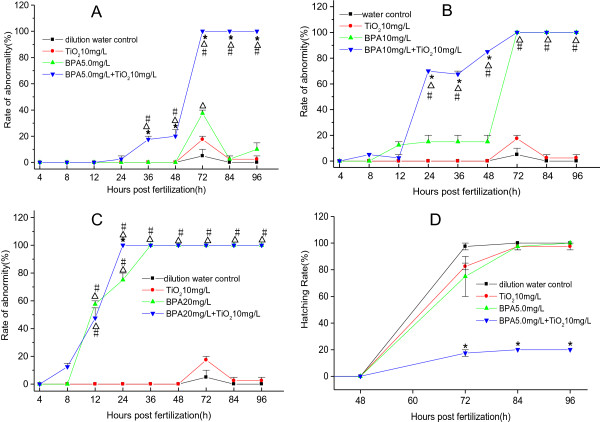
**Rates of abnormality (A-C) and hatching rate (D) of the embryos.** *Significant difference between the BPA alone-exposed and mixture-exposed groups. ^∆^Significant difference compared to the dilution water control. ^#^Significant difference compared to the TiO_2_-NP alone control (chi-square test, *p* < 0.05).

For the rate of abnormalities, the embryos were observed to increase abnormalities after being exposed to the mixture groups at 5, 10, and 20 mg/L of BPA, except for a small reduction at 12 hpf in the mixture-exposed groups at BPA concentrations of 10 and 20 mg/L. Compared to the BPA alone-exposed groups, the durations that abnormality rate elevated significantly were 36 to 96 hpf after the mixture exposure at 5 mg/L BPA, 24 to 48 hpf after the mixture exposure at 10 mg/L of BPA, and 24 hpf after the mixture exposure at 20 mg/L of BPA, respectively (*p* < 0.05) (Figure 7A, B, C).

The combined exposure of BPA and TiO_2_ had an effect on the hatching rate. As shown in Figure 7D, the hatching rate of embryos exposed to the mixture groups at BPA concentrations of 5 mg/L was significantly retarded compared to that of embryos exposed to the BPA alone-exposed groups.

## Discussion

### Regularity for combined toxic effects of TiO_2_-NPs and BPA on zebrafish embryos

**Figure 8:**
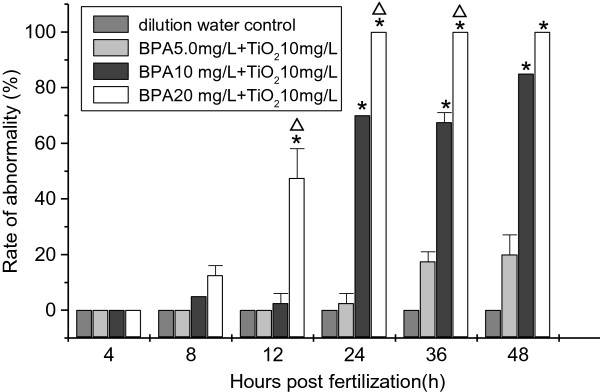
**Rates of abnormality of the embryos.** *Significant difference compared to the BPA 5 mg/L + TiO_2_ 10 mg/L group. ^∆^Significant difference compared to the BPA 10 mg/L + TiO_2_ 10 mg/L group (chi-square test, *p* < 0.05).

In the embryo toxicity tests, the appearances of toxicological effects were different: the embryos were mainly observed to have developmental retardation at 8 to 24 hpf, and then heart sac edema and even death were observed after 36 hpf. With the increasing concentration of BPA in the mixture-exposed groups, the embryos were significantly sensitive at the endpoints of 24 h no spontaneous movement and heart sac edema. Both the percentage of 24 h no spontaneous movement and that of heart sac edema displayed significant increases in the mixture groups at 10 and 20 mg/L of BPA compared to the single BPA groups. Moreover, it could also be found that there was a concentration-dependent effect in the mixture-exposed groups at the endpoints of 24 h no spontaneous movement and heart sac edema. For the abnormality rate, exposure to the mixture groups almost increased the rate of abnormalities compared to the BPA alone-exposed groups and showed a concentration-dependent effect and time-dependent effect. With the increasing doses of BPA (from 5, 10, to 20 mg/L) in the mixture-exposed groups, the abnormality rates elevated at 12, 24, 36, and 48 hpf (Figure 8). All the analyses above suggest that mixture exposure increased the toxicological effects on the zebrafish embryos compared to the BPA alone-exposed groups. There was a concentration-dependent effect in the toxicological endpoints among the mixture-exposed groups.

In addition, it was also found that the significant increases of combined toxic effects compared to the single groups were in connection with the doses of BPA in mixture. For example, compared to the BPA alone-exposed groups, there were no significant differences at 0.5, 1, and 2 mg/L BPA in the mixture-exposed groups, whereas significant differences occurred at 5, 10, and 20 mg/L BPA in the mixture-exposed groups. Moreover, the beginning time of significant difference occurred earlier at the higher dose (20 mg/L BPA) mixture group than at the lower dose (5 and 10 mg/L BPA) mixture groups. At the same time, the duration of significant difference was shorter at the highest dose of BPA mixture group than at the lower dose of BPA mixture groups. For example, compared with BPA alone-exposed groups, the significant increasing abnormalities occurred at 24 hpf in the groups of 20 mg/L BPA mixture and at 36 to 96 hpf in the groups of 5 mg/L BPA mixture. Therefore, we conclude that the combined toxic effects on the development of zebrafish embryos were enhanced significantly within a tested dose range of BPA under the same dose of TiO_2_-NPs.

### The mode of combined action

The combined toxicological effects include additive effects, synergistic effects, potentiation effects, and antagonism effects. In this study, the addition of TiO_2_-NPs powder into individual concentrations of BPA solutions mainly caused increased toxicity as evidenced by decreased survival, increased morphological abnormalities, and delayed embryo hatching. Although the abnormality rates of the mixture-exposed groups at BPA concentrations of 10 and 20 mg/L were lower than those of the corresponding BPA alone-exposed groups at 12 hpf, there were no significant difference between them. Based on these data, we suggest that the mode of action of BPA and TiO_2_-NPs has a synergistic effect.

### Influencing factors of combined toxicological effects

In this study, we evaluated the combined toxicological effects of BPA and TiO_2_-NPs by embryo toxicity testing. Several influencing factors may have caused different combined toxicological effects and are as follows: (1) the dose ratio of BPA to TiO_2_-NPs may have caused differential toxicity and (2) the physical properties of the TiO_2_-NPs, including the particle diameter, degree of dispersion of the suspension, and sedimentation rate.

### The link between the adsorption experiments *in vitro* and the combined toxicological effects *in vivo*

Based on the physical and chemical properties of NMs, it is easy to adsorb chemicals in the environment. Once the chemical is adsorbed, the toxicity effects of NMs on organisms were likely to change. In our study, the adsorption experiments *in vitro* had demonstrated that adsorptive interactions did exist between TiO_2_-NPs and BPA. The zebrafish embryo experimental results confirmed the combined toxic effects and showed mainly increased toxicological effects which were different from the single chemical. The toxicity of the same doses of BPA was enhanced under the existence of TiO_2_-NPs. One reason may be the adsorptive interactions and loading effects of NMs on the organic chemical BPA. The mobility and transport of BPA adsorbed to NMs might be enhanced. We hypothesize that TiO_2_-NPs in combination with BPA could increase BPA bioavailability and uptake into cells and organisms.

However, these results were insufficient to explain the interactions between these two chemicals. The investigation of the interaction of mechanisms for mixtures requires understanding dynamics related to the state of external exposure for the chemicals, toxicokinetics of the chemicals within the organisms, and toxicodynamics of chemicals at the target site. All of these require multidisciplinary tools and techniques [31]. In our future studies, we will examine how the mixtures could affect their bioavailability and uptake into the organism.

## Conclusions

Based on their exceptional physicochemical properties, TiO_2_-NPs are most likely to adsorb other organic contaminants in water. In our study, the *in vitro* adsorption experiments had demonstrated that adsorptive interactions do exist between TiO_2_-NPs and BPA. Data from the zebrafish embryo toxicity test had indicated that combined exposure of the two chemicals increased the toxicological effects with dose dependence. We also suggest that the mode action of BPA and TiO_2_-NPs has a synergistic effect. Moreover, we postulate that concomitant exposure to TiO_2_-NPs and BPA increased BPA bioavailability and uptake into cells and organisms. Further studies are required to understand the mechanisms of interactions of this mixture.

## Competing interests

The authors declare that they have no competing interests.

## Authors’ contributions

JY and CLH carried out the experiments and drafted the manuscript. BCL performed the statistical analysis. HSZ and ZQL participated in the design of the study. ZGX guided this work. All authors read and approved the final manuscript.
